# Supplementary material for: Impact of the surplus distribution principle on the development of agricultural cooperatives in China
Source: PLoS One. 2025 May 21;20(5):e0323876. doi: 10.1371/journal.pone.0323876 (PMC12094751; doi:10.1371/journal.pone.0323876)
Supplement: Attachment — Submitted filename: Response to Reviewers.docx [file pone.0323876.s003.docx]

**Response to Reviewers**

Dear Editors and Reviewers:

Thank you for giving us the opportunity to submit a revised manuscript entitled "A Study on the Impact of the Principle of Surplus Distribution on the Development of peasant Cooperatives in China" for publication in PLOS ONE.We appreciate the time and effort that you and the reviewers dedicated to providing feedback on our manuscript and are grateful for the insightful comments on and valuable improvements to our paper. All suggestions made by you and the reviewers have been incorporated into the manuscript. Those changes are marked in red within the manuscript. Please see below a point-by-point response to your and the reviewers' comments and concerns. The authors welcome further constructive comments if any.

**Response to Associate** **Editor**

**# Comment 1.** Thank you for your contribution to PLOS ONE. After careful consideration, we believe it has merit, but does not currently fully meet PLOS ONE's criteria for publication. We therefore invite you to submit a revised manuscript to address the issues raised during the review process.One of the reviewers suggested only minor changes and was generally satisfied with the paper. However, the second reviewer suggests significant changes and raises a number of questions that you need to address correctly in order to finally consider publishing.

**# Response 1.** Thank you very much for your time and consideration. While we did not receive your specific comments about the article, we appreciate the reviewers for their valuable input. We have carefully considered their suggestions and made the necessary changes to address their concerns. If you have any other feedback or suggestions, we would appreciate it.

**Response to Reviewer 1**

This paper studies the impact of normalized earnings distribution on the development of cooperatives based on the two-way fixed utility model, aiming to evaluate the validity of the surplus distribution method based on transaction income. The results show that the distribution of surplus strictly according to the distributable surplus of more than 60% has a significant negative impact on the scale of members of cooperatives, but has a significant positive impact on cooperative income. This study helps to understand the impact of the principle of surplus distribution on the performance and scale of farmers' cooperatives from the macro level, and provides important policy implications for promoting the standardized construction of farmers' surplus distribution and guiding members to establish a stable interest linkage mechanism with cooperatives.

Here are some comments:

**# Comment 1.** The abstract should answer why your research is necessary? Why are these findings useful and important? The current style of writing has little impact. Although the author emphasizes the important influence of earnings distribution, we cannot get a preliminary definition from the abstract. Is earnings distribution the surplus of unified operation or the surplus of product profit difference? In addition, the summary does not indicate the size of the survey sample.

**# Response 1.** Thank you very much for this great comment!After modification, we indicate in the abstract that the survey data of this paper comes from “*Provincial Panel Data on Cooperative Development and Surplus Distribution in 30 Provinces in China, 2008-2021*”（Line 9–10）. The analysis of the results shows that, theoretically, the surplus distribution method, which is strictly based on the trading return of more than 60% of the distributable surplus, has a significant negative effect on the membership size of cooperatives, while it has a significant positive effect on the income of cooperatives. In terms of practice（Line 18–22）：“*this study helps to understand the impact of surplus distribution principles on the performance and size of farmers' cooperatives at the macro level, and provides important policy insights for promoting the standardization of surplus distribution in farmers' cooperatives and guiding members to establish a stable benefit linkage mechanism with cooperatives*”.In the introduction（Line 62–68）, we add the definition of distributable surplus from China's Law on Farmers' Specialized Cooperatives.

**# Comment 2.** This Cooperative surplus distribution problems in India are indeed worthy of study, and could be raised to the level of the developing countries' presentation in the introduction to enhance the generality of the article's conclusions.

**# Response 2.** Thank you very much for this great comment!In the introduction（Line36–38）, we add a comparison of farmers' professional cooperatives in developing and developed countries and provide an overview of the characteristics of farmers' professional cooperatives in developing countries. At the same time, we enriched the article with a description of the development of farmers' specialized cooperatives in developing countries（Line 38–43）: “*Because of the diverse political, economic, and socio-cultural conditions in each country, the trajectory of cooperative development has been quite different, and the patterns of cooperative development and distribution have shown significant variability*”. Following this, we have selected relevant studies of agricultural cooperatives in India, El Salvador, Peru and Viet Nam with regard to the distribution of earnings or surpluses to enhance the generality of the findings of the article（Line 44–58）.

**# Comment 3.** The literature citations are very old，and some literatures before 2015 do not belong to the important authoritative literatures and should be replaced. may refer：[https://doi.org/10.3390/ijerph19106255.https://doi.org/10.1007/s10668-022-02744-2.https://doi.org/10.3390/agriculture12081145.https://doi.org/10.1016/j.jclepro.2022.135068.](https://doi.org/10.3390/ijerph19106255.https:/doi.org/10.1007/s10668-022-02744-2.https:/doi.org/10.3390/agriculture12081145.https:/doi.org/10.1016/j.jclepro.2022.135068.)

**# Response 3.** Thank you very much for this great comment！In response to your request, we have removed eleven non-authoritative citations and retained only four relatively important pre-2015 citations. At the same time, we have added fifteen post-2015 cited literatures, including four provided by the reviewers, based on adjustments to the content of the article.

**# Comment 4.** In line 97-106, the author argues that no one has studied the effect of surplus distribution on the scale of cooperation and membership dynamics. Generally speaking, no one's research direction may lack significance. The author should elaborate more on why this topic research is meaningful and what reference suggestions it provides for policy making.

**# Response 4.** Thank you very much for this valuable Comment! We have added a discussion of the significance of this study（Line 90–93）: “*This paper aims to narrow these gaps by investigating the impact of the surplus distribution principle, as stipulated in China’s Cooperative Law, on the development of agricultural cooperatives. Specifically, we focus on two key dimensions: the size and income of cooperatives.*” In the third part of the research contribution, we add the implications of this study for policy making:（Line 104–106）: “*This study provides important policy insights for promoting the standardization of surplus distribution in developing countries, and for guiding members to establish a stable mechanism for linking interests with cooperatives.*”.

**# Comment 5.** In the theoretical analysis, the author introduced the concept of property rights system, and spent a lot of space from the company to the cooperative society, which is very good. However, when it comes to the cooperative property rights surplus distribution system, there is a lack of corresponding literature support, and the elaboration is less.

**# Response 5.** Thank you very much for this great comment! First, we add a discourse on the nature of surplus distribution systems and property rights in cooperatives: “*The type of distribution system adopted by cooperatives is essentially related to the issue of surplus control and surplus claim rights in cooperatives”*（Line 134–136）. Secondly, the issue based on cooperative member ownership is added (Line 189–193): “*In a cooperative property rights system, granting ownership to cooperative members may have beneficial incentive effects, but the distribution of benefits and surpluses among members may also lead to free-rider problems within the firm, which can negate the incentive effects of cooperative ownership*.” And we add Kenkel and Bijman's views on the distribution of cooperative surpluses (Line 236–242).

**# Comment 6.** 160 lines of discussion on the size of cooperative members, I suggest that we can draw an optimal size graph, which will be more vivid.

**# Response 6.** Following the reviewers' comments, we have added a plot of the relationship between cooperative size and performance. This relationship graph is an inverted U-shaped curve (Line 190–190).

**# Comment 7.** The formulation of hypothesis 1 is too cumbersome and should be condensed

**# Response 7.** Thank you for pointing this out! In accordance with the reviewer's suggestion, we have simplified hypothesis 1 to read (Line 204–206): “*The principle of surplus distribution on a trade basis will have a dampening effect on the size of cooperatives compared to cooperatives that do not return on a trade basis*”. In addition, in order to maintain the balance between hypotheses 1 and 2, we simplified hypothesis 2 to read: “*The principle of surplus distribution on a trade basis has a catalytic effect on the income of cooperatives compared to cooperatives that do not return on a trade basis*”（Line 244–246）

**# Comment 8.** The endogeneity test part can be advanced.

**# Response 8.** Thank you for your valuable suggestions on our paper, especially your attention to the issue of endogeneity. Based on your feedback, we will further consider using alternative instrumental variables to address the endogeneity problem. Following your suggestion, we will use the cooperative's historical surplus from the previous year as a new instrument to replace the lagged explanatory variable in Section 5.3, in order to effectively resolve the endogeneity issue (Line 410–418): “*To avoid serious endogeneity problems that could have biased and inconsistent effects on the estimated coefficients, this paper takes measures to deal with endogeneity. In this paper, the historical surplus of the cooperative in the previous year was used as an instrumental variable and regression analysis was conducted using two-stage least squares (2SLS) method using the reduced-form data. The regression results are presented in Table 7 and show that the sign and significance level of the coefficients of the parameter estimates of cooperative size and income are unchanged by strictly trading back more than 60% of the distributable surplus, indicating that the results of the benchmark regression are robust.*” We have also updated the results of the endogeneity test, as shown in Table 7 (Line 419).

**# Comment 9.** The author needs to design and write a “Discussion” section, where the author shows how the work advances the field from its current state of knowledge. The similarities, differences and innovative findings of this paper with previous studies. I suggest the authors develop a new section called "Limitations and Future recommendations". Study limitations are the constraints placed on the ability to generalize from the results, to describe applications further to practice.

**# Response 9.** We thank the authors for their interest in the concluding section of the paper! At the suggestion of the authors, the title of section VI has been changed from “*Conclusions and Policy Implications*” to “*Discussion and Policy Implications*”. Furthermore, we have divided section VI into two parts: “*Discussion*” and “*Policy Implications*”. In the second paragraph of the “*Discussion*” section (Line 436), we show that the results of the study provide three research perspectives on the development of cooperative organizations. In the fourth paragraph of the “*Policy Implications*” section, we have added a third policy implication (Line 478-479): “*Thirdly, it is important to strengthen the linkage of interests between cooperatives and their members, and between members and their members, to ensure that members get what they pay for.*”. On this basis, we have added a section 7 entitled “*Limitations and Future Recommendations*” (Line 491) in order to summarize the limitations of this study and suggest future directions for research and practice. In the second paragraph of this section, we describe one of the directions for future research with a real-world example (Line 478-479): “*For example, as the duration of education and training increases, does the productive capacity and willingness of community members to specialize increase as their professional capital accumulation becomes richer?*” Second, in the third paragraph, we also suggest some directions for future research (Line 507–510): “*In the future, the types of cooperatives can be categorized in more detail, and by conducting research on a number of cooperatives of different types, general patterns can be analyzed and whether the principle of surplus distribution has different results for the development of different types of cooperatives can be explored.*”.

**Response to Reviewer 2**

**# Comment 1.** Introduction and Research Hypothesis:According to what I've read in the paper, you wish to assess how surplus affects the "development of farmers' cooperatives." The literature you cited regarding the significance of surplus is very broad and sections 1 and 2.1 provide an overview of it.

**# Response 1.** Thank you very much for your recognition! However, since you did not put forward any specific modification suggestions for this part, it was only partially modified according to the opinions of the first reviewer.

**# Comment 2.** Methodology and Data

**# Comment 2.1.** I suggest to better explain the computation of your main independent variables: “surplus" defined as “number of cooperatives with more than 60% of distributable surplus on a transaction basis/total number of cooperatives (%)” - see Table 1.

**# Response 2.1.** Thank you for your valuable comments, especially regarding the calculation method of the "*surplus*" variable. We greatly appreciate your attention to this detail. Regarding the calculation method you mentioned for the "*surplus*" variable, specifically, this variable represents the proportion of cooperatives in which the surplus allocated exceeds 60% of total cooperatives. We first calculate the surplus allocated by each cooperative in each trading period, then identify how many of these cooperatives allocate more than 60% of their surplus. Finally, we divide the number of cooperatives meeting this criterion by the total number of cooperatives to obtain the percentage of this variable. To ensure that readers can clearly understand the calculation process of this variable, we will further clarify this point in the paper and add the corresponding explanation in Table 1 (Line 306). We sincerely thank you again for your careful review of the paper's details and look forward to your further feedback.

**# Comment 2.2.** I also have a concern about the dependent variable since I believe it could be more appropriate considerer other dependent variables in the equation. "Size" (total number of cooperative members/total number of cooperatives (persons)) is obviously a key variable in the analysis but the Number of Cooperatives is included among the different independent variables” sales; delivery; brand; business; finance" as the denominator. Because of this, I don't think that this represents a good indicator.

**# Response 2.2.** Thank you for your detailed review of our paper, particularly your valuable comments regarding the choice of the dependent variable. Regarding your concern about the dependent variable "*Size*" we believe that, within the current research framework, "*Size*" (the ratio of total cooperative members to the number of cooperatives) is an appropriate key variable. It reflects the scale of cooperatives and serves as a summary of other critical variables when analyzing the overall performance of cooperatives. We understand your concern that "*Number of Cooperatives* " is already considered in the denominator of an independent variable. However, we still believe that the use of "*Cooperative Size*" in our model remains justified, as it independently provides information on the scale of cooperatives and holds its own distinct role in the analysis.

**# Comment 2.3.** Revenue (Total income from cooperatives/total number of cooperatives (10,000RMB)) is a proxy of development, but it could be that costs are very high and therefore this affects development and performance and consequently welfare. I am not familiar with your dataset and the information it contains, but it might be interesting to add other proxies of cooperatives development, depending on the availability of data, for example:

•Sales Growth rate (you included “the sales rate of agricultural products (sales)” as control)

•Sales/N Members (you included “the sales rate of agricultural products (sales)” as control)

•Profit

•Profit/N Members

Or -If available:

•Average Value Added

Of course, considering the lagged independent variables

**# Response 2.3.** Thank you for your valuable feedback on our paper, particularly your suggestions regarding the choice of proxy variables. We greatly appreciate your attention to the details of our research. Regarding the use of "*Revenue*" as a proxy variable for cooperative development, we believe that this variable effectively reflects the overall development level of cooperatives. Revenue, as a comprehensive indicator of cooperative operations and market performance, is a key factor in assessing the economic health of cooperatives. While we understand that costs may also influence cooperative performance, in the context of our research framework, revenue has proven to be the most representative variable for the overall development of cooperatives. Therefore, we consider "*Revenue*" to be a suitable proxy variable. Additionally, regarding the other proxy variables you mentioned, such as sales growth rate, sales per member, profit, etc., we agree that these are indeed valuable variables. However, due to limitations in the current dataset, we have not been able to include all of them. That said, we have accounted for the "*Agricultural Product Sales Rate*" as a control variable. This indicator reflects the percentage of cooperatives that sell more than 80% of their agricultural products, which we believe covers some of the factors you raised. In future research, we will consider expanding the dataset to include additional variables that could further enhance our analysis. Once again, we thank you for your insightful suggestions and look forward to your further feedback. We are committed to optimizing our research based on your valuable input.

**# Comment 2.4.** In addition, given the high correlation between the variables, I suggest adding the correlation matrix in the text.

**# Response 2.4.** Thank you for your valuable suggestions on our paper, especially regarding the inclusion of the correlation matrix. Your feedback has been extremely helpful in enhancing the clarity of our paper and the data analysis section. In response to your suggestion, we have included the correlation matrix between the variables in the paper. We believe this will help readers better understand the relationships among the variables, particularly in cases where there is a high correlation between them. Additionally, we have provided an explanation of these relationships within the main text (Lines 308). Once again, we appreciate your insightful suggestions, and we will fully consider and incorporate your feedback in the revised version of the paper. If you have any further suggestions or questions, we are more than happy to continue the discussion and further improve our research.

**# Comment 3.** Econometric approach

My concern is related to Section 5.3 about the endogeneity. As I read “In this paper, the explanatory variables are lagged by one period as instrumental variables, and regression analyses are conducted using two-stage least squares (2SLS) with the reduced-form data”. I appreciate your efforts to explain the empirical approach, the IV, and other details, but given the issue that you also highlighted, I believe that in this case, an instrument different from lag variable is required to address the endogeneity.

**# Response 3.** Thank you for your valuable suggestions on our paper, particularly your attention to the endogeneity issue in Section 5.3. We greatly appreciate your feedback on the selection of instrumental variables in our model and are eager to improve and refine our analysis methods based on your advice. In response to your feedback, we will further consider using alternative instrumental variables to address the endogeneity problem. Following your suggestion, we will replace the lagged explanatory variable with the cooperative’s historical surplus from the previous year as a new instrumental variable in Section 5.3. This adjustment aims to effectively resolve the endogeneity issue. The revision is as follows (Lines 423–431): “*To avoid serious endogeneity problems that could have biased and inconsistent effects on the estimated coefficients, this paper takes measures to deal with endogeneity. In this paper, the historical surplus of the cooperative in the previous year was used as an instrumental variable and regression analysis was conducted using two-stage least squares (2SLS) method using the reduced-form data. The regression results are presented in Table 7 and show that the sign and significance level of the coefficients of the parameter estimates of cooperative size and income are unchanged by strictly trading back more than 60% of the distributable surplus, indicating that the results of the benchmark regression are robust.*” Additionally, we have updated the results of the endogeneity tests, which are shown in Table 7 (Lines 432–432). Thank you once again for your valuable suggestions. We will continue to refine the paper based on your feedback. If you have any further recommendations or questions, we are happy to continue the discussion and improve the research.

**# Comment 4.** Additional points
• Comparison -from a descriptive point of view -with other countries.
• Compute a map that illustrates China's agricultural cooperative distribution system.
• Think about how the geographical area or regions differ from one another.

**# Response 4.** Thank you very much for your recognition! In the second paragraph of the introduction (Line 36–57), we added the differences in the development of specialized farmers' cooperatives between developing countries, including China, and developed countries. In the third paragraph of the “*Discussion*” section, we have added the differences between geographical regions and the differences between farmers' specialized cooperatives in China and those in other countries (Line 464–470): “*The biggest difference between Chinese farmers' cooperatives and those of other developing countries lies in the heterogeneity of the cooperative membership system. In order to harmonize the interests of capital and labor, China's Law on Farmers' Specialized Cooperatives allows capital to share a portion of the surplus, but not more than 40%, which takes care of capital's interests while allowing laborers to hold the majority of the surplus claim. The findings also provide important insights into the development of agricultural and farmers' cooperatives in developing countries in transition.*”.
