# Supplementary material for: Impact of the surplus distribution principle on the development of agricultural cooperatives in China
Source: PLoS One. 2025 May 21;20(5):e0323876. doi: 10.1371/journal.pone.0323876 (PMC12094751; doi:10.1371/journal.pone.0323876)
Supplement: Attachment — Submitted filename: Response_to_Reviewers.docx [file pone.0323876.s004.docx]

**Response to Reviewers**

Dear Editors and Reviewers:

Thank you very much for giving us the opportunity to submit a revised draft of the manuscript entitled "Impact of the surplus distribution principle on the development of agricultural cooperatives in China " (ID: HSSCOMMS-19456R1) for publication in *PLOS ONE*. We appreciate the time and effort that you and the reviewers dedicated to providing feedback on our manuscript. All suggestions made by you and the reviewers have been incorporated into the manuscript. Please see below a point-by-point response to your and the reviewers' comments and concerns.

**Response to Associate Editor**

**# Comment 1.** I would like you to provide some further discussion about the endogeneity issues involved in the empirical analysis as well as in support of the IV strategy adopted to address those, as suggested by Reviewer #2.

**# Response 1.** Thank you very much for your time and consideration. While we haven't received any comments from you, we appreciate the valuable comments provided by the reviewers. We have carefully considered their suggestions and have made the necessary revisions to address their concerns. If you have any additional feedback or suggestions, we would be grateful to receive them.

**Response to Reviewer 1**

**# Comment 1.** No Response

**# Response 1.** We would like to thank you for your valuable comments, which have significantly improved the quality of the manuscript. Please let us know if there are any errors in the revision of the manuscript. The authors will revise it as soon as possible.

**Response to Reviewer 2**

**# Comment 1.** Thank you for the opportunity to read the manuscript revised version. I appreciated the authors effort in improve the paper contents and I understand the difficult to obtain additional data. However, I feel greater attention should be given to the issue of endogeneity presented by the author in section 5.3. In particular, I would recommend expanding the discussion on the use of instrumental variable and the exclusion restriction, providing additional details and argument on the reason for employing the aforementioned instrument. Finally, I suggest showing the first stage findings.

**# Response 1.** Thank you very much for pointing this out. We have expanded the discussion on the use of the instrumental variable and the exclusion restriction as suggested by the reviewer: "*We select the historical surplus from the previous year as the IV. First, the IV satisfies the relevance condition with core explanatory variables. A cooperative's historical surplus level directly influences management decisions on distribution rules, with cooperatives demonstrating strong past surpluses being more likely to maintain high-proportion transaction volume return policies. Table 7 shows the significant explanatory power of IV in the first-stage regression (F-statistic = 28.98), thus rejecting the weak instrument hypothesis. Second, the IV meets the exclusion restriction. The previous year's surplus primarily affects cooperative development through its impact on current distribution policy selection, with a low likelihood of directly influencing the membership scale or income through alternative mechanisms. Specifically, the economic effects of the historical surplus are absorbed by model-controlled fixed assets and regional and time fixed effects, whereas reputation effects are constrained by short-term fluctuations and captured by brand variables. The two-stage least squares regression results in Table 7 demonstrate that after controlling for endogeneity, the sign and significance level of the estimated coefficients of the core explanatory variables remain robust. Notably, the absolute coefficient values of the core variables increase compared with the baseline regression, suggesting that the ordinary least squares estimates might suffer from downward bias owing to measurement errors or omitted variables. The IV approach not only confirms the robustness of the baseline conclusions but also reveals that conventional regression may underestimate the actual impact of surplus distribution principles.*"

**Table 7. Endogeneity test results**

| Variables | First-stage | Second-stage | |
| --- | --- | --- | --- |
|  | SURPLUS | SIZE | REVENUE |
| IV | 2.024^***^ |  |  |
|  | (0.52) |  |  |
| SURPLUS |  | -9.410^***^ | 7.455^***^ |
|  |  | (-4.18) | (2.30) |
| SALE | 0.036^**^ | 0.578^***^ | 0.078 |
|  | (0.02) | (3.09) | (0.43) |
| SUPPLY | 0.088^***^ | 1.036^***^ | -0.257 |
|  | (0.02) | (3.50) | (-0.83) |
| BRAND | 0.194^*^ | 4.310^***^ | 4.397^***^ |
|  | (0.12) | (4.12) | (4.05) |
| ENTITY | 0.402^***^ | 4.014^***^ | -2.852^*^ |
|  | (0.10) | (3.16) | (-1.92) |
| FINANCE | 0.140 | 1.909^**^ | 0.002 |
|  | (0.09) | (2.50) | (0.01) |
| FARMLAND | 0.444 | -4.828 | 5.657 |
|  | (0.97) | (-0.52) | (0.56) |
| AGRPOP | -0.001 | -0.049 | -0.010 |
|  | (-0.01) | (-0.88) | (-0.13) |
| AGRPRO | -0.359^**^ | -0.557 | 4.307^***^ |
|  | (-0.15) | (-0.45) | (3.10) |
| _cons | 16.285^***^ | 29.553 | 71.606^**^ |
|  | (-4.39) | (1.17) | (2.28) |
| FIXED UTILITY | YES | YES | YES |
| N | 420 | 420 | 420 |
| R^2^ | 0.867 | 0.800 | 0.783 |
| F | 28.98 | 13.09 | 19.13 |

Notes: ***, **, and * denote significance at the 1%, 5%, and 10% levels, respectively; z-values of the statistics are in parentheses.

Thank you again for your valuable suggestions to improve the quality of our manuscript. If there any other modifications we could make, we would like very much to modify them and we really appreciate your help.
